# Artificially regulated synthesis of nanocrystals in live cells

**DOI:** 10.1093/nsr/nwab162

**Published:** 2021-09-09

**Authors:** An-An Liu, En-Ze Sun, Zhi-Gang Wang, Shu-Lin Liu, Dai-Wen Pang

**Affiliations:** State Key Laboratory of Medicinal Chemical Biology, Tianjin Key Laboratory of Biosensing and Molecular Recognition, Frontiers Science Center for New Organic Matter, Research Center for Analytical Sciences, College of Chemistry, School of Medicine, Frontiers Science Center for Cell Responses, Nankai University, Tianjin 300071, China; College of Chemistry and Molecular Sciences, Wuhan University, Wuhan 430072, China; State Key Laboratory of Medicinal Chemical Biology, Tianjin Key Laboratory of Biosensing and Molecular Recognition, Frontiers Science Center for New Organic Matter, Research Center for Analytical Sciences, College of Chemistry, School of Medicine, Frontiers Science Center for Cell Responses, Nankai University, Tianjin 300071, China; State Key Laboratory of Medicinal Chemical Biology, Tianjin Key Laboratory of Biosensing and Molecular Recognition, Frontiers Science Center for New Organic Matter, Research Center for Analytical Sciences, College of Chemistry, School of Medicine, Frontiers Science Center for Cell Responses, Nankai University, Tianjin 300071, China; State Key Laboratory of Medicinal Chemical Biology, Tianjin Key Laboratory of Biosensing and Molecular Recognition, Frontiers Science Center for New Organic Matter, Research Center for Analytical Sciences, College of Chemistry, School of Medicine, Frontiers Science Center for Cell Responses, Nankai University, Tianjin 300071, China

**Keywords:** cell, synthesis, nanocrystal, quantum dot, artificial, metabolism

## Abstract

Live cells, as reservoirs of biochemical reactions, can serve as amazing integrated chemical plants where precursor formation, nucleation and growth of nanocrystals, and functional assembly, can be carried out accurately following an artificial program. It is crucial but challenging to deliberately direct intracellular pathways to synthesize desired nanocrystals that cannot be produced naturally in cells, because the relevant reactions exist in different spatiotemporal dimensions and will never encounter each other spontaneously. This article summarizes the progress in the introduction of inorganic functional nanocrystals into live cells via the ‘artificially regulated space–time-coupled live-cell synthesis’ strategy. We also describe ingenious bio-applications of nanocrystal–cell systems, and quasi-biosynthesis strategies expanded from live-cell synthesis. Artificially regulated live-cell synthesis—which involves the interdisciplinary application of biology, chemistry, nanoscience and medicine—will enable researchers to better exploit the unanticipated potentialities of live cells and open up new directions in synthetic biology.

## Teaser

A strategy of ‘artificially regulated space–time-coupled synthesis’ enables live cells to intracellularly synthesize functional inorganic nanocrystals, opening a new window for synthetic biology.

## INTRODUCTION

In the last decade, cells have been exploited as a powerful tool to accomplish unexpected tasks through artificial regulation. Given the numerous reactive intermediates generated in the sophisticated metabolic networks and the subtle redox balance that supports intracellular homeostasis, cells can function as chemical factories to produce various nanocrystals [1]. Some redox reactions endow the cell with the ability to change the valence of heavy metal ions, which is essential for its survival in stressful/toxic environments [2,3]. Under the pressure of natural selection, some microorganisms have evolved the ability to spontaneously synthesize nanoparticles, or even hierarchical structures. This is known as biomineralization, bioremediation or bioleaching*.* These processes have been incorporated into important strategies to produce minerals, mainly natural products, with marvelous functions [4].

**Figure 1.**
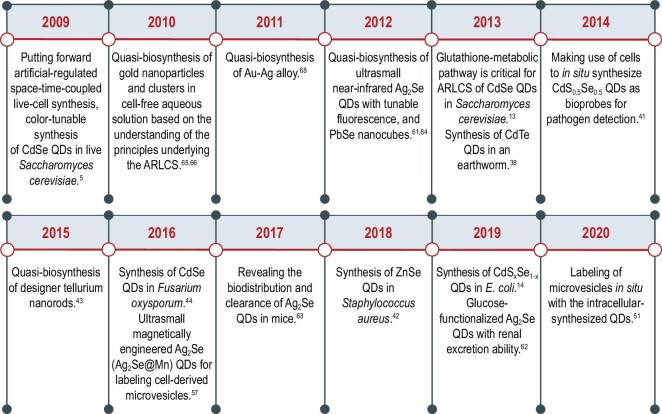
Timeline of key developments in artificially regulated space–time-coupled live-cell synthesis (ARLCS).

However, it is still a great challenge to deliberately direct intracellular reactions and pathways to synthesize desired products that cannot be produced naturally in the cell, in cases where the required reactions exist in different spatiotemporal dimensions and would never coincide spontaneously. To overcome this barrier, we proposed the concept of ‘artificially regulated space–time-coupled live-cell synthesis’ (ARLCS), which means purposefully and precisely coupling a series of intracellular metabolic pathways in an appropriate spatial and temporal sequence to synthesize nanocrystals, such as fluorescent semiconductor quantum dots (QDs), in live cells. Compared with conventional chemical synthesis approaches using toxic organic solvents at elevated temperature and pressure, ARLCS can be effectively controlled in mild (i.e. physiological) conditions, endowing the nanocrystals with inherent biostability and biocompatibility [5,6]. In addition to the specific desired physicochemical properties of the nanocrystals, they are protein encapsulated and water dispersible without needing to resort to additional biofunctionalization processes such as ligand exchange and encapsulation treatment. Thus, ARLCS offers a unique route to synthesizing nanocrystals that are suitable for specific bio-applications. The strategy has subsequently been expanded to synthesize nanocrystals in cell-free systems that mimic intracellular processes in a mild aqueous solution containing enzymes, electrolytes, peptides and coenzymes. The synthesis routes in these quasi-biosynthesis strategies can be elaborately designed based on an understanding of the principles underlying live-cell synthesis of various nanocrystals (Fig. 1).

In this review, we focus on: (i) synthesis of inorganic nanocrystals with desired properties in live cells; (ii) promising and ingenious bio-applications of nanocrystal-synthesizing cells; and (iii) quasi-biosynthesis systems. Finally, we discuss future possibilities and challenges of the live-cell synthesis and cell-free quasi-biosynthesis strategies.

## HOW TO SYNTHESIZE DESIGNER NANOCRYSTALS IN LIVE CELLS

Precursors resulting from cellular metabolism are a prerequisite for nanocrystal synthesis. The reactivity of the precursors, the fed amount of raw chemicals, the feeding ratio and the feeding order are all vitally important to the morphology, size and properties of the product nanocrystals. In conventional chemical synthesis, the precursors are usually prepared separately before the production of nanocrystals, whereas, in ARLCS, the precursors are produced by feeding the cell with suitable chemicals and triggering intended intracellular metabolic pathways. These reactive precursors are mostly complex, unstable and are present in the cell at trace levels. However, by regulating the amount and oxidation state of the chemicals, the time and order of addition, and the incubation time, we can adjust the production and reactivity of the cell-generated precursors.

**Figure 2.**
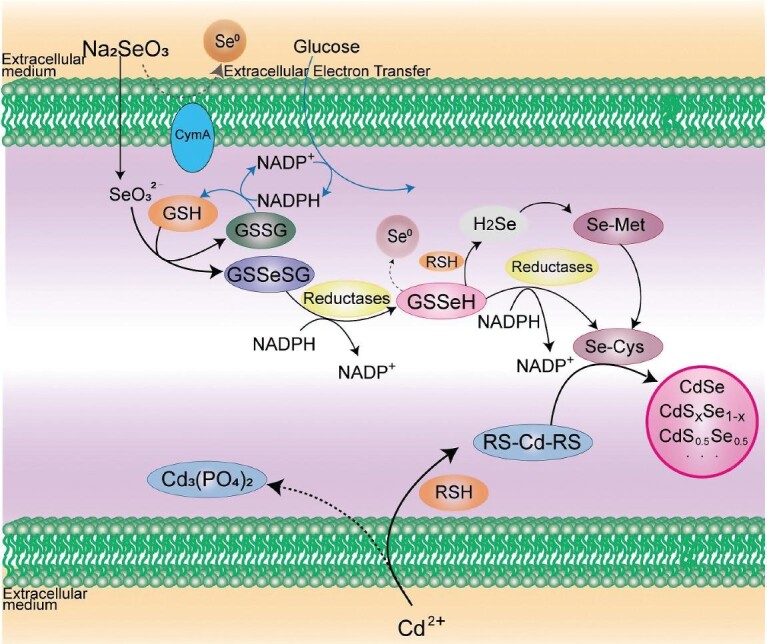
Metabolic pathways in ARLCS of fluorescent quantum dots in *Saccharomyces cerevisiae* and *Escherichia coli.*

In the case of live-cell synthesis of CdSe QDs, it is essential to produce both reactive Se- and Cd-containing precursors at the proper intracellular location and timepoint (Fig. 2). Selenium is a valence-variable element that can exist in multiple organic and inorganic forms. Among these, high-valence Na_2_SeO_3_ [Se(IV)] is generally selected as a selenium source; it can be reduced to selenodiglutathione (GSSeSG) in the intracellular redox environment, driven by reduced thiols (RSH) such as glutathione (GSH) (reaction 1). GSSeSG can be further reduced to unstable low-valence selenium, glutathioselenol (GSSeH), catalyzed by GSH-related enzymes such as glutathione reductase (GR) in the cytoplasm and mitochondria (reaction 2) [5]. GSSeH either spontaneously decomposes into GSH and elemental selenium (Se^0^) or is further reduced to volatile hydrogen selenide (H_2_Se/HSe^−^/Se^2−^) by GSH (reactions 3 and 4) [7]. The downstream metabolites of hydrogen selenide are multiple organoselenium compounds, including selenocysteine (SeCys), L-selenocystine [(Cys-Se)_2_] and selenomethionine (SeMet) (Fig. 2); this has been confirmed in *Saccharomyces cerevisiae* cells by high-performance liquid chromatography coupled with inductively coupled plasma mass spectrometry analysis and the use of selective selenol probes [5,8,9]. The consumption of GSH induces the upregulation of the expression of cysteine-synthesis-related genes, which can promote the conversion of SeMet to SeCys [10]. Unstable low-valence selenium compounds are the reactive Se-containing precursors

 

required for the synthesis of CdSe [5].

(1) \begin{eqnarray*} 4{\rm{GSH}} &+& {\rm{Se}}{{\rm{O}}_3}^{2 - }{\rm{\ }} + {\rm{\ }}2{{\rm{H}}^ + }{\rm{\ }} \to {\rm{\ GSSeSG\ }}\nonumber\\ &+& {\rm{\ GSSG\ }} + {\rm{\ }}3{{\rm{H}}_2}{\rm{O}} \end{eqnarray*}

(2) \begin{eqnarray*} {\rm{GSSeSG\ }} &+& {\rm{\ NADPH\ }} + {\rm{\ }}{{\rm{H}}^ + }{\rm{\ }}\mathop \to \limits^{GR} {\rm{\ \ GSSeH\ }}\nonumber\\ &+& {\rm{\ GSH\ }} + {\rm{\ NAD}}{{\rm{P}}^ + } \end{eqnarray*}

(3) \begin{equation*} {\rm{GSSeH\ }} \to {\rm{\ S}}{{\rm{e}}^0} + {\rm{GSH}} \end{equation*}

(4) \begin{equation*} {\rm{GSSeH}} + {\rm{GSH\ }} \to {\rm{\ }}{{\rm{H}}_2}{\rm{Se}} + {\rm{GSSG}} \end{equation*}

**Figure 3.**
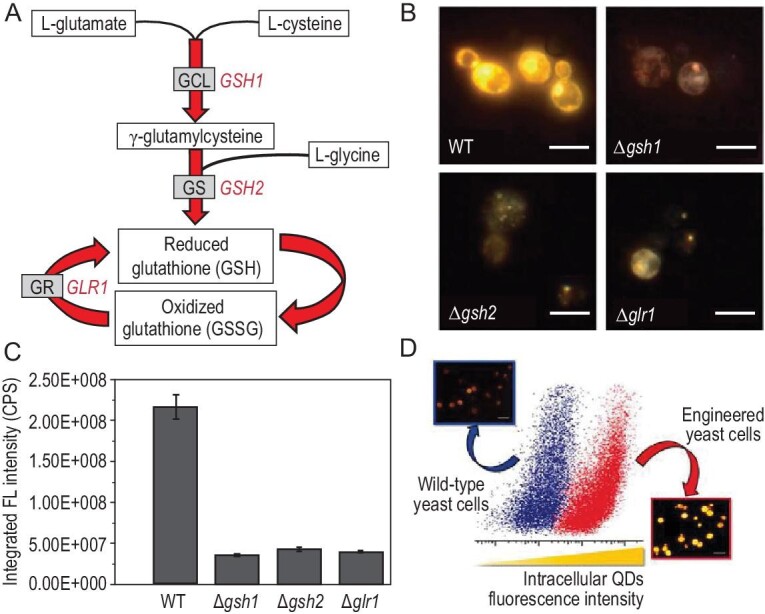
Metabolic pathway of glutathione in *S. cerevisiae* yeast cells, which participates in the ARLCS of CdSe QDs. (A) The formation of γ-glutamylcysteine is the rate-limiting reaction of glutathione synthesis, catalyzed by the γ-glutamylcysteine ligase (GCL, *GSH1*-encoded). The γ-glutamylcysteine is further consumed to produce reduced glutathione (GSH) by reacting with glycine in the presence of catalyst glutathione synthetase (GS, *GSH2*-encoded). The oxidized glutathione (GSSG) is reduced to GSH under the catalysis of the glutathione reductase (GR, *GLR1*-encoded). (B) Fluorescence images (scale bar, 5 *μ*m) and (C) fluorescence intensity of wild-type (WT) and engineered (Δ*gsh1*, Δ*gsh2* and Δ*glr1*) *S. cerevisiae* cells. (D) Flow cytometry measurement and fluorescence images of WT (blue) and engineered (P*_GAL1_*-*GSH1*) *S. cerevisiae* (red) cells after ARLCS of CdSe QDs (scale bar, 10 *μ*m) [13].

As shown in reactions 3 and 4, the metabolite that is formed from GSSeH depends on the GSH concentration. When the level of GSH is moderate, Se^0^ is produced and accumulated, which is a detoxification mechanism in many microorganisms [11]; upon formation of Se^0^, the cells turn red (the color of Se^0^) [12]. A high level of both GSH and reduced nicotinamide adenine dinucleotide phosphate (NADPH) is essential to produce reactive Se-containing precursors (Fig. 3) [13]. In yeast cells, GSH and NADPH can be synthesized with high efficiency during the stationary phase (SP), which is hence usually selected as the period when the Se source is added to cells for ARLCS [5,12]. As well as GSH, other intracellularly generated RSH, including glutaredoxin (GRX) and thioredoxin (TRX), whose expression levels can be significantly elevated by addition of glucose in *Escherichia coli* (*E. coli*) cells, can also act as reducing agents to convert Na_2_SeO_3_ to reactive Se-containing precursors [14].

Particular pathways in certain bacteria can also affect the products of Se reduction. For instance, in *Shewanella oneidensis* MR-1, a widely distributed dissimilatory metal-reducing bacterium, fumarate reductase FccA reduces Na_2_SeO_3_ to Se^0^ in the periplasm [15]. The extracellular electron transfer (EET) ability of this bacterium enables the reduction, which is regulated by the key membrane-anchored protein CymA. Impairing EET by deleting *cym*A gene significantly enhanced the production of CdSe QDs in the cytoplasm, indicating that several intracellular Se reduction pathways can proceed in parallel, leading to distinct products [11]. In *E. coli*, Na_2_SeO_3_ uptake is inhibited by phosphate, and intracellular Se is transformed from Se^0^ to organoselenium compounds when the phosphate level is high. This is mainly attributed to the competitive uptake of phosphate and Na_2_SeO_3_ mediated by the low-affinity phosphate transporter PitA [16].

In addition, a high concentration of Na_2_SeO_3_ (≥10 mM) results in the generation of reactive oxygen species, bringing about oxidative stress, which inhibits the growth of yeast cells [5,17]. Therefore, once the concentration of Na_2_SeO_3_ is too high, a detoxication pathway will be triggered, and Se^0^ becomes the predominant product. To maximize the yield of organoselenium products and hence improve the yield of QDs, the concentration of Na_2_SeO_3_ must be moderate and optimized. Although Na_2_SeO_4_ can also serve as a high-valence Se source, it is seldom employed because of its high toxicity and low transformation efficiency to reactive Se-containing precursors [9,18].

As well as Se, Cd is required for the synthesis of CdSe QDs. Because Cd(II) is very toxic to cells, organisms have evolved several mechanisms to counter Cd toxicity. Some intermediates generated in the detoxification process are suitable Cd-containing precursors for CdSe synthesis in live cells. For instance, in yeast cells, Cd(II) is sequestered by metallothioneins or chelated by GSH because of their high cysteine content [10,19–21]. The generated bis(glutathionato)cadmium [Cd(GS)_2_], a reactive Cd-containing precursor, is subsequently transported to and isolated in vacuoles to achieve detoxification; this transport is regulated by adenosine triphosphate (ATP)-binding cassette transporters [22]. However, once Cd(GS)_2_ is sequestered in the vacuole, there is little Cd-containing precursor left in the cytoplasm, which is unfavorable for the formation of CdSe. In *E. coli*, ingested Cd(II) binds predominantly with phosphate groups and is mainly transformed into Cd_3_(PO_4_)_2_ precipitate to decrease the toxicity of cadmium [14,23]. These pathways for Cd(II) might outcompete the CdSe synthesis pathway [14]. However, glucose addition can enhance the synthesis of NADPH and RSH, favoring competitive binding of Cd with the RSH over phosphate groups, resulting in the production of Cd-containing precursors instead of Cd_3_(PO_4_)_2_ in *E. coli*. It is worth mentioning that GSH/RSH can also act as a sulfur source to participate in the ARLCS of CdSe to generate CdS_x_Se_1−x_ ternary QDs [14] or form CdS spontaneously to overcome the toxicity of Cd [24,25]. Therefore, adding glucose can substantially increase the intracellular formation of CdS_x_Se_1−x_ QDs [14]. Similar to selenium, a high concentration of Cd (≥1 mM) can severely inhibit enzyme activity and cell growth. Thus the concentration of Cd must be moderate and optimized.

The crucial role of GSH-related metabolic pathways in the synthesis of CdSe QDs in yeast cells is evidenced in strains with mutations in GSH metabolism (Fig. 3A). The *gsh1* and *gsh2* genes encode γ-glutamylcysteine ligase and glutathione synthetase, which catalyze the first (rate-limiting) and the second reactions of cellular glutathione synthesis, respectively. Deleting either or both genes stops production of glutathione in cells. Compared with the wild-type, these mutant strains showed a significant decrease in the biosynthetic yield of CdSe QDs [13]. Likewise, treatments with compounds that react with and therefore consume GSH, such as 1-chloro-2,4-dinitrobenzene and buthionine sulfoximine, dramatically decrease the biosynthetic yield of QDs [26]. In comparison, the yield is elevated in engineered *S.cerevisiae* cells in which GSH expression is upregulated by galactose [13]. Moreover, acidic stress is reported to up-regulate the expression of several GSH-related genes including *cysK* (encoding cysteine synthetase) and *gsh2*, which can also enhance the synthetic yield of QDs [27].

As discussed above, there are several pathways working in parallel to generate reactive Se- and Cd-containing precursors, and the Cd compounds tend to be sequestered to reduce toxicity, which means these precursors do not encounter each other in natural physiological conditions. In addition, because of its toxicity, the Cd source, CdCl_2_, can inhibit the reduction of selenium, hence the selenium must be reduced before the addition of CdCl_2_ [5]. QDs exhibit unique size-dependent optical properties due to the quantum confinement effect [28]. Hence, on extending the incubation of selenized *S.cerevisiae* with CdCl_2_ from 10 to 40 h, the diameter of the QDs produced by the ARLCS strategy could be tuned from 2.69 to 6.34 nm, with corresponding emission wavelengths ranging from *ca*.520 to 670 nm [5]. Rational balance and coupling of a series of indispensable but unrelated pathways in appropriate space and time sequences are of vital importance to achieving the ARLCS of QDs [5].

According to the liquid chromatography-mass spectrometry analysis, the proteins intrinsically encapsulated on the intracellularly synthesized QDs mostly function in energy metabolism. Unlike the conventional chemical synthesis, which mostly occurs at ∼200–300°C, ARLCS proceeds in ambient physiological conditions. Thus, it is of significance to investigate the underlying energy driving the reactions [8,14]. ATP is the commonest direct energy resource in live cells. By introducing several ATP-synthesis-deficient strains of yeast cells, ATP is proved to be a pivotal energy resource in ARLCS; it participates in the accumulation of Se-containing precursors, the uptake of Cd and the formation of QDs [8]. The yield of live-cell-synthesized QDs can be enhanced 2-fold in genetically modified cells in which the ATP level is elevated [8].

In 1989, Dameron *et al*. achieved the biosynthesis of CdS QDs by culturing the yeasts *Candida glabrata* and *Schizosaccharomyces pombe* in the presence of cadmium salts [22]. Since then, a broad range of transition metal chalcogenide semiconductor nanocrystals, such as CdS, PbS and ZnS, have been synthesized in different microorganisms [23,29–37]. These nanocrystals are formed following the typical intracellular biomineralization pathways triggered by metal–thiolate polynuclear clusters formed via the interaction between the transition metal and thiolate. This mechanism is different from the concept of ARLCS. On one hand, the biosynthesis of CdS and PbS in these reports generally makes use of thiolate-containing peptides in which sulfur naturally exists in a low oxidation state (S^2−^). The S^2−^ ions can directly bind to metal ions, and generate polynuclear clusters that nanocrystals develop from. In contrast, the low-valence reactive Se in the ARLCS of CdSe is generated from the reduction of high-valence Se in raw chemicals via intracellular metabolic reactions. On the other hand, the CdS or PbS nanocrystals biosynthesized in the typical intracellular biomineralization pathways accumulate in vacuoles, in which the metal precursors are sequestered in metal–γ-glutamyl complexes [29]. However, in the ARLCS of CdSe, multiple metabolic pathways of Se and Cd are spatiotemporally coupled (reactions 1–4). As a result, the Cd-containing precursors generated in the cytoplasm and mitochondria are hijacked by reactive Se-containing precursors and transformed into the desired QDs before isolation in vacuoles can occur [5]. Because of this mechanism, the diameter and corresponding emission wavelength of the QDs can be tuned by the amount of the added raw chemicals and the incubation time, which has never been achieved using the biomineralization strategy. In addition, the growth of the nanocrystals can be controlled (made slow) in live cells, facilitating the crystallization and production of QDs with desired sizes and optical properties.

As well as fungal and bacterial cells, the ARLCS strategy has been extended to earthworms [38]. In 2013, Stürzenbaum *et al.* used the same strategy to expose *Lumbricus rubellus* earthworms to soil spiked with CdCl_2_ and Na_2_TeO_3_ for 11 days, achieving the synthesis of CdTe QDs in the earthworms. Although their proposed synthesis mechanism was similar to the above-mentioned reactions 1–4 (but replacing the Na_2_SeO_3_ with Na_2_TeO_3_), further experimental evidence is necessary to support such a mechanism [38]. Their as-prepared CdTe QDs are 2.3 nm in diameter with a fixed emission maximum at 520 nm. The fluorescence lifetime is only 4.54 ns, which is 20-fold lower than that of the CdSe synthesized in *S.oneidensis* MR-1 cells (99.8 ns) [11,38]. The lifetime, defined by the average time the electron spends in the excited state prior to returning to the ground state, is one of the most important characteristics of a fluorescent nanocrystal. The fluorescent nanocrystals with long lifetimes are preferred in bio-applications. Based on the proposed mechanism of ARLCS, the optical properties of the QDs synthesized in *L.rubellus* earthworms could be enhanced by artificially regulating the amount of added raw chemicals and the incubation time.

The ‘space–time coupling ARLCS strategy’ was first proposed to account for the synthesis of CdSe QDs in *S.cerevisiae* in 2009 [5,8,26,28,39,40], and it has been expanded to the synthesis of various nanocrystals, such as CdTe, ZnSe, CuSe and Te nanorods, in other cell types including *Staphylococcus aureus* [41–43], *Fusarium oxysporum* [44], *E. coli* [45], *S.oneidensis* [11,15,46], *Bacillus licheniformis* [47], *Bacillusamyloliquefaciens* [48], *Rhodotorula mucilaginosa* [49], *Candida utilis* [50], mammalian cells [51], *Tetrahymena pyriformis* [52,53], *Caenorhabditis elegans* [54] and earthworms [38,55], by easily altering the raw chemicals fed to the cells (Fig. 1). As mentioned above, the redox reactions that participate in generating the reactive anion and cation precursors are dependent on the intracellular metabolic network. By elaborately coupling these reactions, supercells containing functional inorganic nanocrystals are created for some ingenious bio-applications.

## WHAT TO DO WITH THE NANOCRYSTAL-CONTAINING CELLS

Despite terrific progress in the synthesis of nanocrystals in live cells, it is still challenging to conveniently and skillfully use the fluorescence properties of intracellular nanocrystals for further application. One straightforward idea is to isolate the synthesized fluorescent nanocrystals from cells by ultrasonication, ultrafiltration and centrifugation. For instance, extracted intracellular-synthesized core-shell QDs (CdS_x_Se_1−x_ core with protein- and phosphate-rich capping synthesized in *E. coli* cells) can be directly used as a sensitive Hg(II) probe based on a Cd(II)–Hg(II) substitution; these QDs exhibit a linear fluorescent response to Hg(II) concentration in the range 1.5–100 nM. Strikingly, in the higher concentration range 0.1–10 μM, Hg(II) can be easily detected by the naked eye once the load of QDs has been raised. Therefore, this is a label-free method for Hg(II) detection, which implies the high potential of live-cell-synthesized QDs for environmental monitoring applications that advance the development of environmental analytical techniques toward higher sustainability [45].

In some cases, the intricate capping proteins of intracellular QDs make the extraction and purification processes much more difficult. Even worse, the laborious and time-consuming extraction is just the first step, because the nanocrystals need to be further engineered with target molecules before application. Unfortunately, all these processes can induce aggregation of the nanocrystals and impair their fluorescence properties. One way to circumvent the above problems is to exert the fluorescence of intracellular nanocrystals *in situ*, without extraction and purification.

**Figure 4.**
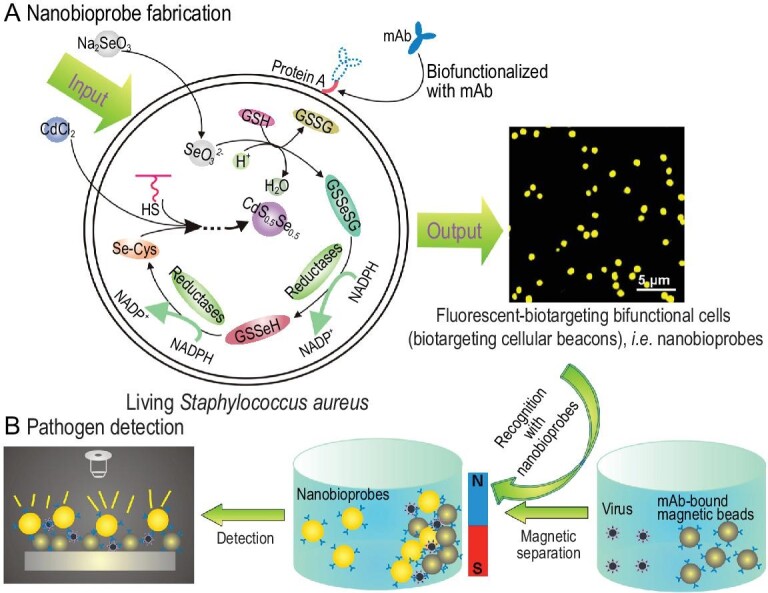
Schematic illustration of the generation and application of nanobioprobes. (A) Fabrication of bioprobes by ARLCS. (B) Using fluorescent-biotargeting bifunctional cells as bioprobes for pathogen detection [41].

For instance, making use of the specific interaction between protein A expressed on the surface of *S.aureus* cells and the Fc fragment domain of antibodies, cells with *in-situ*-synthesized QDs can be readily transformed into nanobioprobes with strong, stable and uniform fluorescence (Fig. 4). This avoids the need for extraction, purification and cell surface modification procedures such as covalent conjugation or genetic and metabolic engineering. Hence, the fluorescence intensity can be maintained to the largest extent, which enhances the sensitivity of detection. Remarkably, this versatile nanobioprobe can be easily adapted to detect diverse pathogens, tumor cells and other biomolecules by simply changing the antibody conjugated to the cell surface. By combining with immunomagnetic beads, the detection limit reached 8.94 ng/mL in H9N2 influenza A virus detection [41].

**Figure 5.**
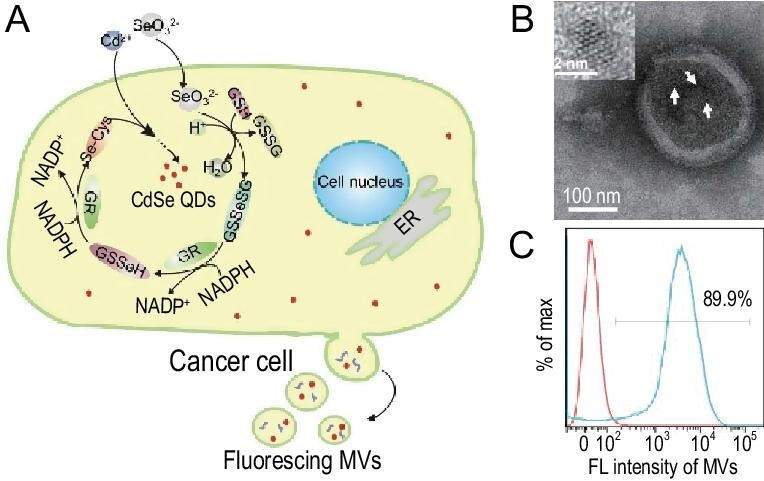
Designer cell-self-implemented labeling of microvesicles (MVs) *in situ* with intracellular-synthesized quantum dots. (A) Schematic illustration of an efficient and biofriendly strategy for one-step labeling of MVs by ARLCS of fluorescent QDs in live MCF-7 cells. (B) *In situ* high-resolution transmission electron microscopy (TEM) image of the QDs in an MV. (C) 90% of MVs can be labeled by the intracellular-synthesized QDs, as measured by flow cytometry analysis [51].

Live-cell-synthesized QDs have also been employed to efficiently label microvesicles (MVs) *in situ* (Fig. 5). Cell-derived MVs can be secreted from almost all types of mammalian cells into the extracellular space, and they play crucial roles in intercellular signaling, communication and transportation. Thus, they can serve as powerful natural carriers for artificial theragnostic agents. By feeding live mammalian cancer cells (MCF-7) with appropriate raw chemicals, CdSe QDs can be synthesized by ARLCS, and MVs derived from the MCF-7 cells can be labeled by the QDs spontaneously in the process of secretion. The whole labeling process skillfully combines the designer synthesis of QDs with mild *in situ* labeling via cell self-implementation. This straightforward method overcomes the problems with conventional post-secretion labeling strategies for MVs such as chemical conjugation, artificial encapsulation and electroporation, which are liable to damage the MV membrane structure and compromise the carrier function [51,56,57].

**Figure 6.**
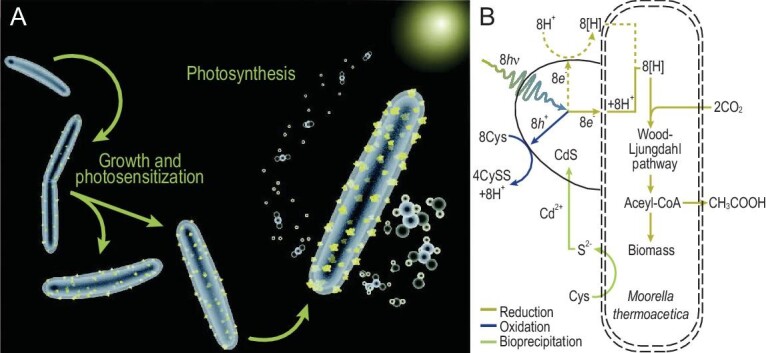
*Moorella thermoacetica*–CdS reaction schemes. (A) In the *M. thermoacetica*–CdS hybrid system, the photogenerated electrons generated from CdS nanoparticles synthesized by *M. thermoacetica* can drive the photosynthesis of acetic acid from CO_2_. (B) Possible photosynthetic mechanism of the *M. thermoacetica*–CdS system [58].

QDs have superior extinction properties, therefore, intracellular-synthesized QDs can act as light harvesters to transform non-photosynthetic cells into an artificial photosynthesis system that produces acetic acid from carbon dioxide. The synthesis of CdS QDs by *Moorella thermoacetica*, a non-photosynthetic bacterium, was triggered by the addition of Cd(II) and cysteine as cadmium and sulfur sources, respectively. The cell can use photogenerated electrons from illuminated CdS nanoparticles to drive a form of photosynthesis (Fig. 6). The absorption of a photon by CdS produces an electron and hole pair. The electron generates a reducing equivalent that is passed into the Wood–Ljungdahl pathway to synthesize acetic acid from CO_2_. Therefore, the QDs also serve as an electron and energy relay in this system [58,59]. In addition, the photogenerated electrons can in turn facilitate the reduction of Na_2_SeO_3_, and consequently favor new QD synthesis [52].

## QUASI-BIOSYNTHESIS SYSTEMS EXPANDED FROM THE ARLCS

As discussed in the last section, it is difficult to purify the QD products from the intricate intracellular environment. Therefore, it is necessary to develop methods that retain the green characteristics of live-cell synthesis whilst avoiding extraction and purification procedures. In the live-cell synthesis route of CdSe, the NADPH/GR system and GSH play important roles in maintaining the reducing environment required for production of the necessary Cd- and Se-containing precursors [5,7,10,13]. Inspired by the principles of ARLCS, we created a cell-free quasi-biological synthesis system containing GSH, NADPH and GR, which is simple compared with the intricate environment in live cells. These bioactive agents can reduce metal ions *in vitro* and produce reactive precursors for QD synthesis, although they have seldom been used in the chemical synthesis of nanomaterials so far because of the lower reactivity of the produced precursors. Using this quasi-biological synthesis system, our group successfully synthesized ultrasmall (sub-3 nm) near-infrared (NIR) fluorescent and water dispersible Ag_2_Se QDs at 90°C [60].

As in ARLCS, the crucial point of quasi-biological synthesis is to obtain Ag- and Se-containing precursors in appropriate oxidation states. With the aid of GSH, NADPH and GR, SeO_3_^2−^ is reduced to low-valence GSSeH, as in the similar process that occurs in live cells in ARLCS. GSSeH, which can react with metal ions such as Ag(I), is predesigned as the Se-containing precursor (reactions 1 and 2). The intended processes of Na_2_SeO_3_ reduction have been evidenced, because all the intermediate products, including GSH, GSSG (oxidized GSH), GSSeSG, and GSSeH, were detected by high-performance liquid chromatography-mass spectrometry. To generate an appropriate Ag-containing precursor, alanine (Ala) is chosen as a stabilizer because it can form Ag(I)–Ala complex and also act as a ligand to stabilize the generated Ag_2_Se QDs. The diameter of the synthesized Ag_2_Se QDs can be precisely controlled by tuning the molar ratio of Ag-containing precursor to Se-containing precursor. Thereby, the photoluminescence (PL) emission peak can be tuned from 700 to 820 nm, corresponding to QD diameters from 1.5 to 2.4 nm [60]. These Ag_2_Se QDs show a strong and efficient cathodic electrogenerated chemiluminescence (ECL) signal on a glassy carbon electrode with K_2_S_2_O_8_ as co-reactant in aqueous solution. The ECL spectrum of the Ag_2_Se QDs exhibited a peak at 695 nm, consistent with the peak in the PL spectrum of Ag_2_Se QD solution, indicating that Ag_2_Se QDs had no deep surface traps that usually impaired the fluorescent properties of QDs [61].

Furthermore, once the synthesized Ag_2_Se QDs are functionalized by glucose (Glc–Ag_2_Se QDs), they demonstrate high uptake in almost all types of cancer cell; this has been applied to *in vivo* long-term tumor imaging. The fluorescence of Glc–Ag_2_Se QDs from the targeted tumor can be observed for at least 7 days, indicating the outstanding *in vivo* stability of the QDs [62]. More importantly, because the Glc–Ag_2_Se QDs are ultrasmall, this probe can be excreted via the kidneys without significant long-term accumulation in organs, which is also favorable for *in vivo* imaging applications [62,63]. The biosafety of the Ag_2_Se QDs has been demonstrated by pathological analysis, blood biochemical analysis and body weights in which the QDs exhibit no appreciable *in vivo* toxicity [63]. All these excellent features mean that the Ag_2_Se QDs have great potential for future clinical use in tumor imaging.

**Figure 7.**
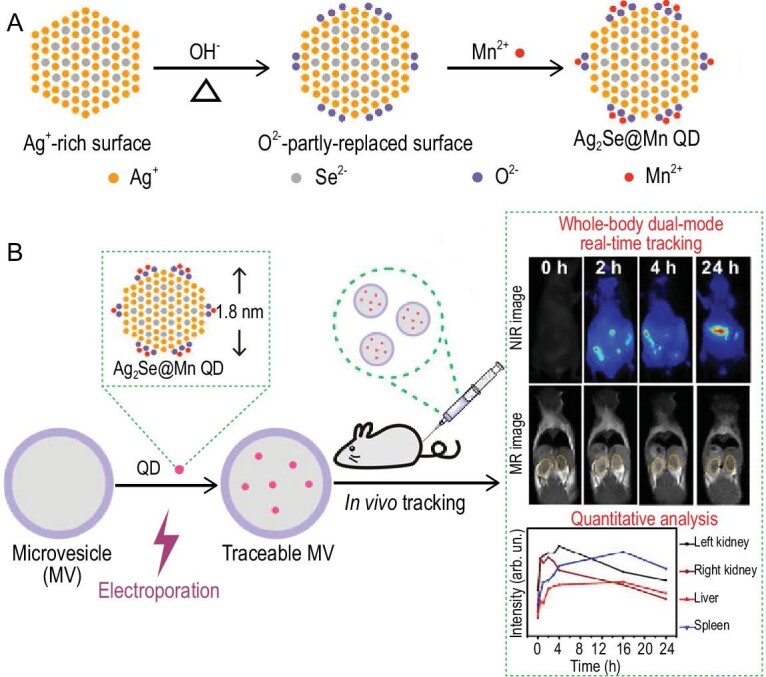
Preparation and application of Ag_2_Se@Mn QDs. (A) Fabrication of the Ag_2_Se@Mn QDs by controlling the reaction of Mn^2+^ with Ag_2_Se nanocrystals pre-treated in NaOH solution. (B) Labeling of cell-derived MVs with Ag_2_Se@Mn QDs by electroporation, and high-resolution dual-mode *in vivo* imaging [57].

The surface of these ultrasmall Ag_2_Se QDs is Ag(I) rich, so it can be transformed into partial O^2−^-terminated by NaOH treatment. This surface engineering facilitates Mn(II) doping via strong coordination with the surface O^2−^, generating ultrasmall water dispersible Ag_2_Se@Mn QDs. The resultant QDs possess high NIR fluorescence quantum yield (13.2%) and longitudinal relaxivity (12.87 mM^−1^ s^−1^). This longitudinal relaxivity is almost four times higher than that of the commercial magnetic resonance imaging contrast agent Gd-diethylenetriaminepentacetate (Gd-DTPA) (Fig. 7) [57]. The ultrasmall size of the Ag_2_Se@Mn QDs enables them to be directly and efficiently loaded into MVs by electroporation, instantly and reliably conferring both NIR fluorescence and magnetic resonance traceability on MVs. The complementary imaging capabilities of the Ag_2_Se@Mn QDs have enabled long-term dual-mode *in vivo* tracking of MVs with high resolution, by which the dynamic biodistribution of MVs has been revealed in a real-time and *insitu* quantitative manner [57].

**Figure 8.**
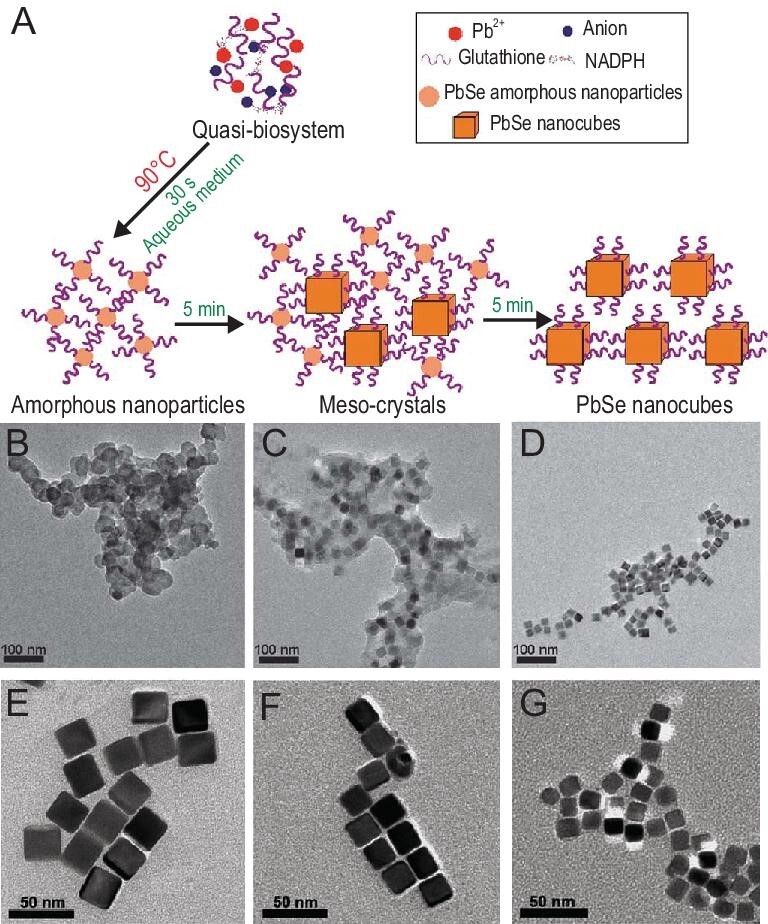
Controllable synthesis of PbSe nanocubes in the aqueous phase using a quasi-biosystem. (A) Schematic illustration. (B–D) TEM images of the mesoscopic transformation of PbSe through a non-classical crystallization process. (E–G) TEM images of PbSe nanocubes synthesized at different initial Pb : Se molar ratios (1 : 1, 1 : 2.5 and 1 : 5) [64].

Similarly, by coupling reduction of Na_2_SeO_3_ with detoxification of Pb(II) in the quasi-biosystem, monodispersed PbSe nanocubes were synthesized in aqueous solution with controllable sizes (Fig. 8) [64]. The crystallization mechanism is that the amorphous precursors are transformed into mesocrystals as intermediates and finally to nanocubes, suggesting that a non-classical crystallization occurred during the formation of the PbSe nanocubes [64]. Using the similar reduction pathways of TeO_3_^2−^, Te nanorods with uniform and tunable lengths (ranging from 10 to 200 nm) can be synthesized as we expected [46]. The molar extinction coefficients are 1.54 × 10^9^ (absorption at 549 nm) and 8.06 × 10^8^ M^−1^ cm^−1^ (absorption at 410 nm) for Te nanorods, respectively, which is comparable to gold nanorods with similar lengths, indicating that Te nanorods may serve as potential photothermal materials in tumor therapy [46].

The mild quasi-biosynthesis system can also be employed to synthesize noble-metal (i.e. gold and silver) clusters and nanoparticles. Compared with conventional methods, this system slows down the reduction of the Au-containing precursors by using a bioreducing agent, NADPH, instead of a strong reducing agent such as NaBH_4_. NADPH molecules initially interact with Au(I) ions via electrostatic force and then reduce them to gold nanoclusters (AuNCs) [65]. Owing to the relatively slow rate of biomimetic reduction, the pH-dependent reduction potential of the reducing agent, and the favorable structure of the capping molecules, we have successfully realized kinetically controlled formation of gold clusters and nanoparticles in mild conditions. The resulting glutathione-capped gold clusters consist of ∼60 Au atoms (Au_60_NCs) with 1.3-nm diameter. Wheat germ agglutinin (WGA)-capped gold clusters consist of 25 Au atoms (Au_25_NCs) with 1.2-nm diameter [66,67]. Generally, such small metal cores are energetically unstable in solution, and tend to aggregate to lower their specific surface energies. However, in the case of quasi-biosynthesis of Au_60_NCs, the abundant GSH molecules capping the surface of the Au cores via strong Au–S bonds provide the product with high stability in aqueous solution. WGA was selected to stabilize the Au_25_NCs via Au–S bonds as it is rich in cysteine residues. Matrix-assisted laser desorption ionization-time of flight (MALDI-TOF) mass spectrometry results indicate that each Au_25_NC is stabilized by only one subunit of WGA [67]. Additionally, the slow production rate of the Au clusters led to separate clusters dispersing in solution, further diminishing the possibility of aggregation [66]. By adjusting the concentration of NADPH, which is easy, the size of the gold nanoparticles (AuNPs) capped with glutathione can be tuned from 6.1 to 12.6 nm with good stability in aqueous solution even in the presence of a high salt concentration [65]. Furthermore, when Au(III) and Ag(I) ions are introduced into the system simultaneously, uniform sub-5 nm Au–Ag alloy NPs tightly capped by NADPH molecules are synthesized [68].

The quasi-biosynthesis strategy can be used to synthesize nanocrystals of interest in a cell-free system, based on an understanding of metabolic pathways. This strategy opens a new avenue for controllable, facile and efficient synthesis of designer nanocrystals for diverse industrial and biomedical applications.

## SUMMARY AND FUTURE PERSPECTIVES

Live cells, as reservoirs of biochemical reactions, can serve as amazing integrated chemical plants for the synthesis of nanocrystals, where precursor formation, nucleation and growth of nanocrystals, as well as functional assembly, can be controlled accurately following an artificial program. Since 2009, by artificially coupling a series of intracellular redox reactions in an appropriate spatiotemporal sequence, various inorganic semiconductor QDs and other nanomaterials have been successfully synthesized in bacterial, fungal and mammalian cells. Inspired by these systems, a cell-free quasi-biosynthesis strategy that simplifies the regulation of intracellular reactions has been developed to produce a variety of nanocrystals in mild conditions, further verifying, strengthening and expanding the methodology for ARLCS.

Generally, the properties of elements in the same group of the periodic table are relatively similar; therefore, multiple elements in the same family may share common intracellular metabolic pathways, which can potentially be used to synthesize different nanocrystals in live cells as well as in cell-free quasi-biological systems. Unfortunately, because of the complexity of intracellular metabolic networks, the deduced practicable pathways have so far focused only on the reduction of chalcogenides (including Se and Te). Thus, the metabolic pathways that have been employed so far in ARLCS are merely the tip of the iceberg. In addition, the nanocrystals synthesized by ARLCS are generally distributed in the cytoplasm, and it is difficult to manipulate the synthesis location in the cell. With this review, we hope to intrigue more researchers to explore new strategies and mechanisms for producing diverse multifunctional crystals and even intricate heteronanostructures and hierarchical structures at desired locations and times.

Besides the metabolic networks, various intracellular biomolecules also play irreplaceable roles in ARLCS. Many biomolecules participate in metabolic reactions and regulate the nucleation and growth processes of nanocrystals. Some biomolecules act as ligands, stabilizing the nanoparticles, and may also provide unique features, endowing the inorganic–biological hybrid systems with potent properties. Therefore, it is necessary to explore the type and function of the biomolecule(s) on the surface of the nanocrystals and investigate the interface of the nanocrystals and biomolecules, but this is very challenging, and it has been largely ignored to date.

Generally, nanoparticles need to be isolated and purified before characterization. However, in the ARLCS system, the laborious and time-consuming extraction process can induce the aggregation of nanoparticles and impair their optical properties. Therefore, *in situ* measurements are required to characterize the size, shape, elemental composition and fluorescence properties of the nanocrystals. Although some powerful approaches, such as electron microscopy, fluorescence spectroscopy, Raman spectroscopy and X-ray absorption near-edge structure spectroscopy are useful for *in situ* studies, it is still difficult to obtain effective and well-defined results [5,11,69]. Studies of live-cell-synthesized nanocrystals, including the surface ligands for stabilizing them, the type and function of encapsulating proteins, and related underlying mechanisms, are still limited by current methodologies.

Compared with chemical synthesis, it is much easier to controllably produce nanocrystals on a large scale by ARLCS, as the number of cells (reactors) can be amplified exponentially and simply by cell culture.

The concept described here is a synthesis route complementary to chemical synthesis, meeting the need to produce materials that cannot be realized by chemical synthesis. It will enable researchers to better exploit the potentials of live cells and allow for new approaches in synthetic biology via the interdisciplinary application of biology, chemistry, medicine and nanoscience.

## References

[1] Li M , Hou F, Wu T et al. Recent advances of metabolic engineering strategies in natural isoprenoid production using cell factories. Nat Prod Rep 2020; 37: 80–99. 10.1039/C9NP00016J 31073570

[2] Korbekandi H , Iravani S, Abbasi S. Production of nanoparticles using organisms. Crit Rev Biotechnol 2009; 29: 279−306. 10.3109/07388550903062462 19929319

[3] Dhillon GS , Brar SK, Kaur S et al. Green approach for nanoparticle biosynthesis by fungi: current trends and applications. Crit Rev Biotechnol 2012; 32: 49−73. 10.3109/07388551.2010.550568 21696293

[4] Yao S , Jin B, Liu Z et al. Biomineralization: from material tactics to biological strategy. Adv Mater 2017; 29: 1605903. 10.1002/adma.201605903 28229486

[5] Cui R , Liu HH, Xie HY et al. Living yeast cells as a controllable biosynthesizer for fluorescent quantum dots. Adv Funct Mater 2009; 19: 2359−64. 10.1002/adfm.200801492

[6] Zhou J , Yang Y, Zhang CY et al. Toward biocompatible semiconductor quantum dots: from biosynthesis and bioconjugation to biomedical application. Chem Rev 2015; 115: 11669−717. 10.1021/acs.chemrev.5b00049 26446443

[7] Tarze A , Dauplais M, Grigoras I et al. Extracellular production of hydrogen selenide accounts for thiol-assisted toxicity of selenite against *Saccharomyces cerevisiae*. J Biol Chem 2007; 282: 875967. 10.1074/jbc.M610078200 17261587

[8] Zhang R , Shao M, Han X et al. ATP synthesis in the energy metabolism pathway: a new perspective for manipulating CdSe quantum dots biosynthesized in *Saccharomyces cerevisiae*. Int J Nanomedicine 2017; 12: 3865−79. 10.2147/IJN.S132719 28579774 PMC5446969

[9] Zhang B , Ge C, Yao J et al. Selective selenol fluorescent probes: design, synthesis, structural determinants, and biological applications. J Am Chem Soc 2015; 137: 757−69. 10.1021/ja5099676 25562612

[10] Shao M , Zhang R, Wang C et al. Living cell synthesis of CdSe quantum dots: manipulation based on the transformation mechanism of intracellular Se-precursors. Nano Res 2018; 11: 2498−511. 10.1007/s12274-017-1873-z

[11] Tian LJ , Li WW, Zhu TT et al. Directed biofabrication of nanoparticles through regulating extracellular electron transfer. J Am Chem Soc 2017; 139: 12149−52. 10.1021/jacs.7b07460 28825808

[12] Wu SM , Su YL, Liang RR et al. Crucial factors in biosynthesis of fluorescent CdSe quantum dots in *Saccharomyces**cerevisiae*. RSC Adv 2015; 5: 79184−91. 10.1039/C5RA13011E

[13] Li Y , Cui R, Zhang P et al. Mechanism-oriented controllability of intracellular quantum dots formation: the role of glutathione metabolic pathway. ACS Nano 2013; 7: 2240−8. 10.1021/nn305346a 23398777

[14] Tian LJ , Min Y, Li WW et al. Substrate metabolism-driven assembly of high-quality CdS_x_Se_1-x_ quantum dots in *Escherichia coli*: molecular mechanisms and bioimaging application. ACS Nano 2019; 13: 5841−51. 10.1021/acsnano.9b01581 30969107

[15] Li DB , Cheng YY, Wu C et al. Selenite reduction by *Shewanella**oneidensis* MR-1 is mediated by fumarate reductase in periplasm. Sci Rep 2014; 4: 3735. 10.1038/srep03735 24435070 PMC3894562

[16] Zhu TT , Tian LJ, Yu HQ. Phosphate-suppressed selenite biotransformation by *Escherichia**coli*. Environ Sci Technol 2020; 54: 10713−21. 10.1021/acs.est.0c02175 32786571

[17] Weekley CM , Harris HH. Which form is that? The importance of selenium speciation and metabolism in the prevention and treatment of disease. Chem Soc Rev 2013; 42: 8870−94. 10.1039/c3cs60272a 24030774

[18] Rahman A , Lin J, Jaramillo FE et al. In vivo biosynthesis of inorganic nanomaterials using eukaryotes—a review. Molecules 2020; 25: 3246. 10.3390/molecules25143246 PMC7397067 32708767

[19] Coyle P , Philcox JC, Carey LC et al. Metallothionein: the multipurpose protein. Cell Mol Life Sci 2002; 59: 627−47. 10.1007/s00018-002-8454-2 12022471 PMC11337511

[20] Delalande O , Desvaux H, Godat E et al. Cadmium-glutathione solution structures provide new insights into heavy metal detoxification. FEBS J 2010; 277: 5086−96. 10.1111/j.1742-4658.2010.07913.x 21078121

[21] Li LL , Cui YH, Chen JJ et al. Roles of glutathione and L-cysteine in the biomimetic green synthesis of CdSe quantum dots. Front Env Sci Eng 2017; 11: 7. 10.1007/s11783-017-0948-0

[22] Li ZS , Lu YP, Zhen RG et al. A new pathway for vacuolar cadmium sequestration in *Saccharomyces**cerevisiae*: YCF1-catalyzed transport of bis(glutathionato)cadmium. Proc Natl Acad Sci USA 1997; 94: 42−7. 10.1073/pnas.94.1.42 8990158 PMC19233

[23] Ulloa G , Quezada CP, Araneda M et al. Phosphate favors the biosynthesis of CdS quantum dots in acidithiobacillus thiooxidans ATCC 19703 by improving metal uptake and tolerance. Front Microbiol 2018; 9: 234. 10.3389/fmicb.2018.00234 29515535 PMC5826283

[24] Dameron CT , Reese RN, Mehra RK et al. Biosynthesis of cadmium sulphide quantum semiconductor crystallites. Nature 1989; 338: 596−7. 10.1038/338596a0

[25] Kowshik M , Deshmukh N, Vogel W et al. Microbial synthesis of semiconductor CdS nanoparticles, their characterization, and their use in the fabrication of an ideal diode. Biotechnol Bioeng 2002; 78: 583−8. 10.1002/bit.10233 12115128

[26] Brooks J , Lefebvre DD. Optimization of conditions for cadmium selenide quantum dot biosynthesis in *Saccharomyces cerevisiae*. Appl Microbiol Biotechnol 2017; 101: 2735−45. 10.1007/s00253-016-8056-9 28004154

[27] Tian LJ , Li WW, Zhu TT et al. Acid-stimulated bioassembly of high-performance quantum dots in *Escherichia**coli*. J Mater Chem A 2019; 7: 18480−7. 10.1039/C9TA06136C

[28] Bao HF , Hao N, Yang YX et al. Biosynthesis of biocompatible cadmium telluride quantum dots using yeast cells. Nano Res 2010; 3: 481−9. 10.1007/s12274-010-0008-6

[29] Kowshik M , Vogel W, Urban J et al. Microbial synthesis of semiconductor PbS nanocrystallites. Adv Mater 2002; 14: 815−8. 10.1002/1521-4095(20020605)14:11<815::AID-ADMA815>3.0.CO;2-K

[30] Spangler LC , Lu L, Kiely CJ et al. Biomineralization of PbS and PbS-CdS core-shell nanocrystals and their application in quantum dot sensitized solar cells. J Mater Chem A 2016; 4: 6107−15. 10.1039/C5TA10534J

[31] Sandana Mala JG , Rose C. Facile production of ZnS quantum dot nanoparticles by *Saccharomyces**cerevisiae* MTCC 2918. J Biotechnol 2014; 170: 73−8. 10.1016/j.jbiotec.2013.11.017 24316439

[32] Kang SH , Bozhilov KN, Myung NV et al. Microbial synthesis of CdS nanocrystals in genetically engineered *E**. coli*. Angew Chem Int Ed 2008; 47: 5186−9. 10.1002/anie.200705806 18512860

[33] Dunleavy R , Lu L, Kiely CJ et al. Single-enzyme biomineralization of cadmium sulfide nanocrystals with controlled optical properties. Proc Natl Acad Sci USA 2016; 113: 5275−80. 10.1073/pnas.1523633113 27118834 PMC4868489

[34] Bruna N , Collao B, Tello A et al. Synthesis of salt-stable fluorescent nanoparticles (quantum dots) by polyextremophile halophilic bacteria. Sci Rep 2019; 9: 1953. 10.1038/s41598-018-38330-8 30760793 PMC6374371

[35] Labrenz M , Druschel GK, Thomsen-Ebert T et al. Formation of sphalerite (ZnS) deposits in natural biofilms of sulfate-reducing bacteria. Science 2000; 290: 1744−7. 10.1126/science.290.5497.1744 11099408

[36] Yan ZY , Du QQ, Qian J et al. Eco-friendly intracellular biosynthesis of CdS quantum dots without changing *Escherichia**coli*’s antibiotic resistance. Enzyme Microb Technol 2017; 96: 96−102. 10.1016/j.enzmictec.2016.09.017 27871390

[37] El-Baz AF , Sorour NM, Shetaia YM. *Trichosporon* *jirovecii*-mediated synthesis of cadmium sulfide nanoparticles. J Basic Microbiol 2016; 56: 520−30. 10.1002/jobm.201500275 26467054

[38] Sturzenbaum SR , Hockner M, Panneerselvam A et al. Biosynthesis of luminescent quantum dots in an earthworm. Nat Nanotechnol 2013; 8: 57−60. 10.1038/nnano.2012.232 23263722

[39] Luo QY , Lin Y, Li Y et al. Nanomechanical analysis of yeast cells in CdSe quantum dot biosynthesis. Small 2014; 10: 699−704. 10.1002/smll.201301940 24130060

[40] Sur VP , Kominkova M, Buchtova Z et al. CdSe QD biosynthesis in yeast using tryptone-enriched media and their conjugation with a peptide hecate for bacterial detection and killing. Nanomaterials (Basel) 2019; 9: 1463. 10.3390/nano9101463 PMC6835635 31623115

[41] Xiong LH , Cui R, Zhang ZL et al. Uniform fluorescent nanobioprobes for pathogen detection. ACS Nano 2014; 8: 5116−24. 10.1021/nn501174g 24779675 PMC4182866

[42] Zhang YN , Yang LL, Tu JW et al. Live-cell synthesis of ZnSe quantum dots in *Staphylococcus**aureus*. Chem J Chin Univ 2018; 39: 1158−63.

[43] Xiong LH , Cui R, Zhang ZL et al. Harnessing intracellular biochemical pathways for in vitro synthesis of designer tellurium nanorods. Small 2015; 11: 5416−22. 10.1002/smll.201500816 26313741 PMC6352974

[44] Yamaguchi T , Tsuruda Y, Furukawa T et al. Synthesis of CdSe quantum dots using *Fusarium**oxysporum*. Materials (Basel) 2016; 9: 855. 10.3390/ma9100855 PMC5456586 28773975

[45] Tian LJ , Min Y, Wang XM et al. Biogenic quantum dots for sensitive, label-free detection of mercury ions. ACS Appl Bio Mater 2019; 2: 2661−7. 10.1021/acsabm.9b00331 35030720

[46] Wang XM , Huang L, Wang YJ et al. Highly efficient near-infrared photothermal antibacterial membrane with incorporated biogenic CuSe nanoparticles. Chem Eng J 2021; 405: 126711. 10.1016/j.cej.2020.126711

[47] Lin TY , Lian ZJ, Yao CX et al. Rapid biosynthesis of fluorescent CdSe QDs in *Bacillus**licheniformis* and correlative bacterial antibiotic change assess during the process. Luminescence 2021; 36: 621−30. 10.1002/bio.3980 33171522

[48] Yan ZY , Yao CX, Wan DY et al. A sensitive and simple method for detecting Cu^2+^ in plasma using fluorescent *B**acillus**amyloliquefaciens* containing intracellularly biosynthesized CdSe quantum dots. Enzyme Microb Technol 2018; 119: 37−44. 10.1016/j.enzmictec.2018.08.009 30243385

[49] Cao K , Chen MM, Chang FY et al. The biosynthesis of cadmium selenide quantum dots by *R**hodotorula**mucilaginosa* PA-1 for photocatalysis. Biochem Eng J 2020; 156: 107497. 10.1016/j.bej.2020.107497

[50] Tian LJ , Zhou NQ, Liu XW et al. A sustainable biogenic route to synthesize quantum dots with tunable fluorescence properties for live cell imaging. Biochem Eng J 2017; 124: 130−7. 10.1016/j.bej.2017.05.011

[51] Xiong LH , Tu JW, Zhang YN et al. Designer cell-self-implemented labeling of microvesicles in situ with the intracellular-synthesized quantum dots. Sci China Chem 2020; 63: 448−53. 10.1007/s11426-019-9697-2

[52] Cui YH , Tian LJ, Li WW et al. Solar-energy-facilitated CdS_x_Se_1-x_ quantum dot bio-assembly in *Escherichia coli* and *T**etrahymena**pyriformis*. J Mater Chem A 2019; 7: 6205−12. 10.1039/C9TA00822E

[53] Cui YH , Li LL, Tian LJ et al. Synthesis of CdS_1-X_Se_X_ quantum dots in a protozoa *Tetrahymena**pyriformis*. Appl Microbiol Biotechnol 2019; 103: 973−80. 10.1007/s00253-018-9499-y 30417309

[54] Li LL , Cui YH, Lu LY et al. Selenium stimulates cadmium detoxification in *C**aenorhabditis**elegans* through thiols-mediated nanoparticles formation and secretion. Environ Sci Technol 2019; 53: 2344−52. 10.1021/acs.est.8b04200 30735361

[55] Talaeeshoar F , Delavari HH, Poursalehi R et al. Can earthworms biosynthesize highly luminescent quantum dots? Luminescence 2018; 33: 850−4. 10.1002/bio.3481 29687574

[56] Smyth T , Petrova K, Payton NM et al. Surface functionalization of exosomes using click chemistry. Bioconjug Chem 2014; 25: 1777−84. 10.1021/bc500291r 25220352 PMC4198107

[57] Zhao JY , Chen G, Gu YP et al. Ultrasmall magnetically engineered Ag_2_Se quantum dots for instant efficient labeling and whole-body high-resolution multimodal real-time tracking of cell-derived microvesicles. J Am Chem Soc 2016; 138: 1893−903. 10.1021/jacs.5b10340 26804745

[58] Sakimoto KK , Wong AB, Yang PD. Self-photosensitization of nonphotosynthetic bacteria for solar-to-chemical production. Science 2016; 351: 74−7. 10.1126/science.aad3317 26721997

[59] Kornienko N , Sakimoto KK, Herlihy DM et al. Spectroscopic elucidation of energy transfer in hybrid inorganic-biological organisms for solar-to-chemical production. Proc Natl Acad Sci USA 2016; 113: 11750−5. 10.1073/pnas.1610554113 27698140 PMC5081607

[60] Gu YP , Cui R, Zhang ZL et al. Ultrasmall near-infrared Ag_2_Se quantum dots with tunable fluorescence for in vivo imaging. J Am Chem Soc 2012; 134: 79−82. 10.1021/ja2089553 22148738

[61] Cui R , Gu YP, Bao L et al. Near-infrared electrogenerated chemiluminescence of ultrasmall Ag_2_Se quantum dots for the detection of dopamine. Anal Chem 2012; 84: 8932−5. 10.1021/ac301835f 23046454

[62] Ge XL , Huang B, Zhang ZL et al. Glucose-functionalized near-infrared Ag_2_Se quantum dots with renal excretion ability for long-term in vivo tumor imaging. J Mater Chem B 2019; 7: 5782−8. 10.1039/C9TB01112A 31482937

[63] Ge XL , Zhang ZL, Xie ZX et al. Revealing the biodistribution and clearance of Ag_2_Se near-infrared quantum dots in mice. New J Chem 2017; 41: 12721−5. 10.1039/C7NJ02126G

[64] Cui R , Gu YP, Zhang ZL et al. Controllable synthesis of PbSe nanocubes in aqueous phase using a quasi-biosystem. J Mater Chem 2012; 22: 3713−6. 10.1039/c2jm15691a

[65] Cui R , Zhang MX, Tian ZQ et al. Intermediate-dominated controllable biomimetic synthesis of gold nanoparticles in a quasi-biological system. Nanoscale 2010; 2: 2120−5. 10.1039/c0nr00193g 20820640

[66] Zhang MX , Cui R, Tian ZQ et al. Kinetics-controlled formation of gold clusters using a quasi-biological system. Adv Funct Mater 2010; 20: 3673−7. 10.1002/adfm.201001185

[67] Zhao JY , Cui R, Zhang ZL et al. Cytotoxicity of nucleus-targeting fluorescent gold nanoclusters. Nanoscale 2014; 6: 13126−34. 10.1039/C4NR04227A 25250903

[68] Zhang MX , Cui R, Zhao JY et al. Synthesis of sub-5 nm Au-Ag alloy nanoparticles using bio-reducing agent in aqueous solution. J Mater Chem 2011; 21: 17080−2. 10.1039/c1jm13120f

[69] Li D , Nielsen MH, Lee JR et al. Direction-specific interactions control crystal growth by oriented attachment. Science 2012; 336: 1014−8. 10.1126/science.1219643 22628650

